# Clinical Correlates of Deliberate Self-Harm Among Migrant Trauma-Affected Subgroups

**DOI:** 10.3389/fpsyt.2021.529361

**Published:** 2021-09-22

**Authors:** Francesca Baralla, Martina Ventura, Nikolay Negay, Anteo Di Napoli, Alessio Petrelli, Concetta Mirisola, Marco Sarchiapone

**Affiliations:** ^1^Department of Medicine and Health Sciences, University of Molise, Campobasso, Italy; ^2^National Institute for Health, Migration and Poverty, Rome, Italy; ^3^Department of Psychiatry and Narcology of Asfendiyarov, Kazakh National Medical University, Almaty, Kazakhstan

**Keywords:** migration trauma exposure, deliberate self-harm behavior, post-traumatic stress (PTS), resilience, non-suicidal self-injury (NSSI)

## Abstract

**Introduction:** Studies have shown that exposure to potentially traumatic events (PTEs) during the migration process has serious consequences on mental health. Migrants with a history of PTEs are more likely to inflict deliberate self-harm (DSH), a spectrum of behavior that includes non-suicidal self-injury (NSSI). With reference to a nonclinical sample of trauma-exposed migrants, this study aims to explore the prevalence of DSH and to assess the association with sociodemographic characteristics and clinical conditions, with particular attention to post-traumatic stress disorder (PTSD) symptoms, resilience capabilities, and feelings of hopelessness.

**Methods:** A sample of migrants underwent a baseline evaluation at an outpatient department of the National Institute for Health, Migration and Poverty (INMP). Migrants with a history of torture, rape, or other severe forms of psychological, physical, or sexual violence were invited to undergo screening at the Institute's Mental Health Unit. Trauma-exposed migrants completed a series of self-report questionnaires that assessed the presence of PTSD, depression, anxiety, suicidal ideation, resilience, and feelings of hopelessness, in addition to DSH. Univariate and multivariate log-binomial regression models were used to test the association of age and clinical characteristic of migrants with DSH. Prevalence ratio (PR) with 95% confidence intervals (95% CI) and *p*-values were estimated.

**Results:** A total of 169 migrants (76.9% males), aged between 18 and 68 years, *M* = 28.93; *SD* = 8.77), were selected. Of the sample, 26.6% were frequently engaging in some form of DSH, and 30.2% were diagnosed with at least one trauma-related disorder. DSH behaviors were most common in single and unemployed migrants as well as in subjects with post-traumatic stress symptoms, feelings of anxiety, hopelessness, low capability of resilience, and suicidal ideation. Taking into account age and hopelessness, we found that PTSD and low resilience capabilities were associated with a higher risk of DSH [PR adj: 2.21; 95% CI: (1.30–3.75) and PR adj: 2.32; 95% CI: (1.16–4.62), respectively].

**Conclusion:** Given the association between trauma exposure and DSH among migrants, exploring the presence of DSH behavior within the immigrant community is crucial for the implementation of measures to develop intervention in a clinical setting.

## Introduction

Deliberate self-harm (DSH) refers to the intentional direct (e.g., destruction of one's own body tissue without suicidal intent) and indirect damage to an individual's body (e.g., severe substance abuse, overdosing, or ingestion of sharp implements), including suicidal behavior (1). Common forms of DSH (2) result in an alteration or damage to body tissue and can refer to multiple methods (3), including skin cutting, burning, scratching, banging or hitting of body parts, and interfering with wound healing (4, 5).

Self-harm is related to psychiatric and personality disorders (6–8) and is particularly widespread, especially in new social and relational contexts, like virtual environments (8).

Self-harm is the most important risk factor for suicide (9) irrespective of the extent, the type, or the motive behind the suicidal intent and therefore may also include suicidal ideation (SUI) (10). SUI is widely acknowledged to be a major risk factor for suicide and seems to be particularly prevalent among first- and second-generation migrants (11).

There are data that show also the association between self-harm and suicide attempt (SA) (12–14). Literature has shown that patients with suicidal intent constitute a more severe group, and self-injuring patients with SUI differ from patients who have not attempted suicide, in terms of greater severity of psychopathology in the former (15).

Non-suicidal self-injury (NSSI), which was proposed as a new diagnosis in the *Diagnostic and Statistical Manual of Mental Disorders* in 2013 (5th ed.) (16), is a prevalent behavioral problem associated nevertheless with poor outcomes and reduced life expectancy (17). Both NSSI and SA are distinct behavioral phenomena that often co-occur within individuals and form a continuum of self-damage that can be related to suicidal behavior (18, 19). Specifically, more recent findings (20) have shown that NSSI increases the risk of transitioning from suicide ideation to a SA.

Some studies have suggested that trauma-related symptoms may play an important role in the development and maintenance of self-harm. As underlined by Ford and Gomez (21), a large body of studies have highlighted the mediating effect of trauma on acts of self-harm. Recently, Sami and Hallaq (22) documented that self-harm is frequently a sequela of prolonged exposure to emotional and physical violence, and post-traumatic stress disorder (PTSD) increases the risk of engaging in self-injurious behaviors (SIBs). Given the important mechanistic role of trauma symptomatology in SIB [e.g., (23)], considering in addition the functional role of SIB in a person's ability to cope with trauma, self-injury has been considered as an effective yet maladaptive strategy to alter one's internal feelings and to alleviate, manage, or eliminate negative emotions and feelings of tension (24).

There has been a long-standing interest in the relationship between the exposure to potentially traumatic events (PTEs) and SIB among migrants [e.g., (1, 25, 26)]. The condition of migration is a potential risk factor for engaging in self-harming behaviors (27), especially among minors (28), by virtue of their higher vulnerability to different pre-migratory, migratory, and post-migratory stress factors, which increase the risk of mental health (29). Ethnic and racial variations in the rates of self-harm have been observed worldwide, with higher prevalence reported among Asian British males and Black females (30). Individuals who have higher levels of ethnic identity and sense of belonging would be less likely to engage in NSSI (31), while migrants with a complex PTSD, exposed to multiple and chronic trauma, especially in interpersonal contexts have been found to present with more severe anger, aggression, and self-harm (26). Among ethnic minorities, self-harm represents one of the most important predictor factors for suicide behaviors (32). The high prevalence of self-harm and suicidal behavior among migrants may therefore be considered as a consequence of the traumatogenic nature of stressful events that can occur in the migrant's country of origin, as well as in their host country (33). Fleeing from warfare and persecution may be considered as a series of traumatic events that can occur pre-, peri-, and post-migration and that may differ in their intensity and duration (34). The variation in rates of post-traumatic psychopathology is related to a variety of factors, specifically to the nature and the intensity of the cumulative PTEs that migrants experience. Studies have shown the effect of “systemic trauma” (23) faced by immigrant populations, which is related to experiences of violence, loss, oppression, and displacement (21). However, very few studies investigated the ethnic and cultural meanings and functions of self-harm in voluntary or forced migrants, in particular taking into account the specifics of displacement and the complex characteristics of trauma in the context of asylum seekers.

The development of intervention to reduce the psychological burden of migrants would be aided by a better understanding of what affects PTSD. In particular, in this study, we took into consideration the construct of hopelessness that has received growing attention in the fields of SIB. Hopelessness can be defined as a trans-diagnostic psychological construct, characterized by rigid and persistently negative expectations about the future and a helplessness to challenge such thoughts (35). It is a phenomenon that it is still understudied in migrant populations. Recent findings (36) suggest that potentially hopelessness can develop into a worsening decline of mental health that can lead to increased self-harm and suicide.

Another key issue, typically related to the migration process and regarded as a “protective” factor, is resilience (37). Resilience is the ability to positively cope with adverse situations and to maintain positive outcomes in the complex interplay between risk and vulnerability factors (38–40). Emerging data suggest that migration research could benefit from the use of a strengths-based approach, including the resilience construct, for a more thorough understanding of migrant experiences (41, 42). Indeed, it has been argued that resilience represents an essential element of epidemiological and prevention research, which aims to promote wellbeing and improve mental health in migrants (43). This is due to the impact that resilience has on how migrants adapt to the migration process and the acculturation experiences in their host countries (44, 45).

Depression and anxiety are psychiatric disorders frequently observed in general populations and are reported to be highly prevalent among migrants, both voluntary migrants and asylum seekers (46). In particular, first-generation migrants reported considerably more depression, generalized anxiety, and panic attacks in the past 4 weeks and SUI than did second-generation migrants (11). Regarding asylum seekers, significant differences in depression and anxiety scores on psychometric instruments among migrants with a history of detention vs. those without were observed (47).

Moreover, a wide range of determinants, connected to migrant's application for asylum, such as uncertainty regarding the outcome, slowness of the procedures, and social isolation, can increase anxiety and depression, representing trigger factors for self-harm (8).

Gaining a better evaluation of self-injury behavior (NSSI and SA), which has been demonstrated to have an association with PTSD [e.g., (48)], is a necessary step in helping healthcare providers to identify and intervene and thus reduce the psychological burden of trauma-exposed migrants. Research on the presence of DSH, related or not to SA, in vulnerable groups such as migrants is required and necessary in order to formulate preventative measures in clinical and treatment settings.

The current study aims to explore the frequency of DSH and document the prevalence of PTSD, depression, and anxiety in a nonclinical convenience subgroup of trauma-affected migrants. It also aims to evaluate the effect of PTSD, resilience capabilities, and feeling of hopelessness on DSH. We hypothesized that PTSD would be associated with an engagement in DSH, while low resilience and feelings of hopelessness would likely act as negative factors that interfere with the adaptation process.

## Methods

### Participants

This study involved PTE-exposed migrants who were selected from consecutive admission to the National Institute for Health, Migration and Poverty (INMP) Ambulatory Care Unit between 2017 and 2018. The research was approved by the institutional review board (IRB) of the National Institutes of Health (Prot. PRE/17). Following admission to the Ambulatory Care Unit of INMP, migrants with a history of torture, rape, or other severe forms of psychological, physical, or sexual violence were approached for participation. Migrants were invited to undergo screening at the Mental Health Unit of INMP, which led to clinical–diagnostic assessments. Migrants involved in the study signed a form consenting to their inclusion in clinical research and received information about voluntary participation, confidentiality, and protection of personal data. All participants were also informed about mental health services and counseling activities, which they could access if need be. Current psychiatric diagnoses were established using clinical interviewing procedures similar to the Structured Clinical Interview for DSM-5 disorders (SCID-5-CV) (49) conducted by trained and supervised clinical research assistants. The screening interview was divided into multiple modules covering background information like demographic characteristics, lifestyle and behavior, trauma history, lifetime history of mental health, and family history of mental disorders. Participants also completed a series of self-report questionnaires, including those described in the Measurements section of this paper, which were randomly ordered to mitigate for order effects.

### Measurements

Migrants completed a self-report questionnaire that assessed exposure to adverse experiences relating to the migration process and/or a trauma history. Furthermore, the presence of the following was ascertained: PTSD, depression and anxiety, resilience, and feelings of hopelessness, DSH, SA and SUI. The research protocol was built *ad hoc* with the aim of gathering relevant information about the migrants' clinical features. After clinical assessment, the participants were asked to complete the following self-administered questionnaires and scales.

#### Measure of Potentially Traumatic Events

To obtain information about the type of PTEs that had occurred during an individual's lifetime, and/or during the migration process, we used the Harvard Trauma Questionnaire (HTQ) (50). Section Introduction of the HTQ is a checklist with 17 items developed as a cross-culturally valid instrument to measure the kind and variety of PTEs. Examples of items are lack of food and water, loss of a loved one, rape, torture, brainwashing, imprisonment, and combat situations. As in a previous study (51), the reporting format was modified, removing the options “witnessed” and “heard about.” The response format allowed participants to indicate when the event had happened; and time periods were coded as in infancy/pre-migration (I/PM) and/or adulthood/during migration (A/DM).

#### Deliberate Self-Harm Inventory

The six-item questionnaire (52) was a modified and shortened version of the Deliberate Self-Harm Inventory (DSHI) (5) that assesses lifetime history of self-harm behavior using a 4-point Likert scale (never; one to two times; three to four times; and five or more times). This inventory is based on the definition of DSH (5) as a direct destruction or alteration of body tissue, without conscious suicidal intent. DSHI measures lifetime presence and frequency of the following self-injury behaviors: self-cutting, self-burning, self-punching, self-scratching, self-carving, self-biting, and self-banging (head and/or other body parts), as well as preventing wounds from healing and skin damage by other methods. In this study, consistent with past studies (52), four indicators of DSH were created. First, in relation to the “history of DSH,” a score of “1” was given to participants who reported a history of DSH behavior. This binary variable was created to indicate a positive screening result for DSH and to identify migrants who may need targeted interventions. For the second variable, “frequency of DSH,” a score of “1” was given to participants who reported having engaged in DSH behavior three or more times (frequent DSH), while a score of “0” was given to participants who reported engaging in DSH behavior twice or less (infrequent DSH). In relation to DSH methods, in line with past studies [e.g., (52)], the third variable DSH-type behavior was defined as a “cutting type” or “non-cutting type”: the “cutting type” consisted of behaviors related to the first, second, third, and fourth items in the DSHI (e.g., “ever intentionally cut wrist, arms, or other area(s) of body, or stuck sharp objects into skin such as needles, pins, staples”), while other forms of DSH were classified as the “non-cutting type” (e.g., “ever intentionally banged your head or punched yourself to the extent that you caused a bruise to appear”). Finally, the variables relating to the participants who reported having harmed themselves so severely as to have warranted hospitalization (“hospitalization” = 1 vs. “no hospitalization” = 0) were dichotomized.

#### Measure of Self-Destructive Behaviors

To determine a lifetime history and/or recent episodes of other self-destructive behaviors, such as SAs and substance use, respondents were asked to report both their lifetime and past year use of drugs (marijuana, cocaine, heroin, inhalants, methamphetamine, and hallucinogens), alcohol, and episodes of attempted suicide, and/or a family history of attempted suicide and/or suicide. Participants responded to each question with a “yes” (=1) or “no” (=0), and answers were classified according to whether or not they reported substance use behaviors, SAs, and/or a family history of SAs. In the case of positive responses related to previous SA(s), to ensure that the answers to (“Have you tried to take your own life?”) were correctly understood, clinicians explained to participants that questions are aimed to assess behavior related to the goal of taking one's own life. Positive answers were identified as emergency cases and referred to a psychiatrist for further evaluation and targeted interventions.

#### Beck Scale for Suicidal Ideation

This is a 19-item scale that assesses a person's current intensity of thoughts, behaviors, and plans to commit suicide (53). A self-reporting version of the scale was introduced by Beck et al. (54). Each item consists of three alternatives that describe different intensities of SUI, which are rated on a 3-point scale from 0 to 2. Participants are instructed to choose the particular statement of each group that is most applicable to them. Total scores are calculated by summing the 19 ratings and can range from 0 to 38, with higher values indicating a greater risk of suicide. Beck and Steer (55) do not distinguish between different degrees of suicidal risk. In line with previous studies (56), very low total scores can be associated with an elevated risk of suicide, and we used the scores ≥2 of the screening part (items 1–5) to identify participants with SUI (SUI = 1 vs. NO SUI = 0). Migrants with positive answers on the Beck Scale for Suicidal Ideation (BSSI) were referred to the medical staff of INMP for further evaluation.

#### Posttraumatic Stress Disorder Checklist for DSM-5

This is a self-report measure that assesses the 20 DSM-5 symptoms of PTSD in the updated version of the PTSD Checklist (PCL). The PCL-5 (57) contains 20 items rated on a 5-point Likert-type scale (available online at: www.ptsd.va.gov), with scores ranging from “not at all” (0) to “extremely” (4), resulting in a symptom severity score ranging from 0 to 80. Factor analysis identified four factors related to the DSM-5 model of PTSD; and they included re-experiencing (RE), hyperarousal (HY), avoidance (AV), and negative feelings (NF), with a three-dimensional factor solution related to the DSM-5 definition. DSM-5 symptom cluster severity scores can be obtained by summing the scores for the items within a given cluster (cluster B = from 1 to 5; cluster C = 6 and 7; cluster D = from 8 to 14; cluster E = from 15 to 20). According to Lang et al. (58), in this study, an overall cutoff score of 33 (≥) was used to indicate the presence of post-traumatic stress symptoms.

#### Zung Self-Rating Depression Scale

This is a self-administered 20-item survey (59) that is used in a variety of settings as a screening tool, covering the common affective, psychological, and somatic characteristics of depression. Each question is framed in terms of positive and negative statements, and each item is scored on a scale ranging from 1 to 4 points (from “a little of the time” to “most of the time”); hence, overall scores range from 25 to 100: scores from 25 to 49 indicate a *normal range*; 50–59, *mild depression*; 60–69, *moderate depression*; and 70 and above, *severe depression*. The scores provide indicative ranges for depression severity. In this study, a cutoff score of 50 (≥) was used to indicate the presence of depressive symptoms.

#### Zung Self-Rating Anxiety Scale

A short self-administered 20-item version of the scale (60), covering both affective and somatic symptoms, focuses on the most common general anxiety disorders. Each response is rated on a 1- to 4-point scale, from “none or little of the time” to “most of the time.” There are 20 questions with 15 increasing anxiety level questions and five decreasing anxiety level questions. Overall scores range from 20 to 80: scores from 20 to 44 indicate a *normal range*; from 45 to 59, *mild anxiety*; from 60 to 74, *moderate anxiety*; and from 75 and above, *severe anxiety*. In this study, a cutoff score of 45 (≥) was used to indicate the presence of anxiety-related symptomatology.

#### Connor-Davidson Resilience Scale 2

This is an abbreviated two-item version of the rating scale for the assessment of resilience, created to reduce administration time (61). The two items of the 25-item Connor-Davidson Resilience (CD-RISC) scale (62) used in this case were item 1 (“Able to adapt to change”) and item 8 (“Tend to bounce back after illness or hardship”). The CD-RISC2 sufficiently represents the original measure, and the CD-RISC2 can be used in place of the 25-item CD-RISC. Test–retest reliability analysis, convergent validity, and divergent validity demonstrated significant correlation (ranging from *r* = 0.27 to 0.66) with both the 25-item CD-RISC version and the individual items of the CD-RISC. In this study, a cutoff score of 5.5 (≤) was used to indicate participants with lower resilience resources (named “Lower resilience”) (61).

#### Beck Hopelessness Scale

This scale (63) includes 20 items that are answered as true or false; and total scores can range from 0 to 20. Cronbach's alpha coefficient of the scale in the general population ranged from 0.82 to 0.93 (64). This scale evaluates three major aspects of hopelessness: an individual's feelings about the future, the loss of motivation, and future expectations. The Beck Hopelessness Scale (BHS) results were dichotomized, using a cutoff score of 9, to differentiate hopeless (1 = presence) from not hopeless subjects (65).

### Data Analysis

A descriptive analysis of the sample was carried out. Categorical variables are presented as absolute numbers (n) and percentage frequencies (%). Sociodemographic and clinical characteristic of the study population were described. The frequencies of PTEs and the period of occurrence (I/PM and/or A/DM) were evaluated to better characterize the migrants included in the sample. In order to evaluate relationships between continuous clinical variables, the correlation matrix was calculated.

The outcome under study was defined as a dichotomous variable, using the information “history of DSH” during a lifetime (1 or more episode) scored by the DSHI. The distribution of DSH behavior was analyzed in the entire sample and for different sociodemographic and clinical characteristics. Differences between categories were assessed using chi-square tests or Fisher's exact test, as appropriate.

In order to evaluate determinants of DSH, in a first step, separate univariate analyses were performed for DSH outcome and all the variables of interest, by using log-binomial regression models. In particular age, anxiety, PTSD, depression, SUI, hopelessness, and lower resilience were tested; prevalence ratios (PRs) with 95% confidence intervals (95% CI) and *p*-values were estimated. Then, we would like to investigate the joint role of age, PTSD, low resilience, and hopelessness on DSH. We first investigated the presence of interactions between hopelessness and other three covariates, but no statistically significant results were found. Finally, multivariate log-binomial regression models were performed to investigate the effect of these covariates on DSH. Two models were run, without and with hopelessness, in order to evaluate the mediating role of this factor on PTSD and low resilience. For all analyses, statistical significance was predetermined at *p* < 0.05, 0.01, or 0.001. Moreover, we also evaluated multicollinearity, to exclude the possibility that correlation between covariates could influence the model results. To this purpose we calculated the variance inflation factor (VIF) and the eigenvalues obtained through a principal component analysis conducted on the matrix of the covariates. The empirical and joint evaluation of the maximum value of VIF, of the absence of very small eigenvalues and of low condition-index values, suggests that problems of multicollinearity can be considered negligible and do not affect the stability of the parameters estimation.

All analyses were performed using SAS® System version 9.3 (66).

## Results

The sample included 169 migrants with permanent residency in Italy aged between 18 and 68, *M* = 28.9; *SD* = 8.77), selected from consecutive admission to the Mental Health Unit of INMP. Table 1 presents the sociodemographic and clinical characteristics of the sample. Respondents were mainly males (76.9%), and 80.5% were young adults (aged 18–35 years); most of them were single (73.4%) and unemployed (77.5%), and about a half had a lower or upper secondary education (49.1%). The 78.7% migrated from Africa (mostly from Western Africa, 67.5%) and 21.9% <1 year ago. Of the migrants, 30.2% had at least one trauma-related disorder, 22.5% at least one mood disorder, and 11.8% at least one substance use disorder. Anxiety disorders and adjustment disorder were both present in 7.1% of the study population. Among participants for whom data were available, 18 (10.7%) declared a family history of mental disorders.

**Table 1:** Sociodemographic and clinical characteristics of the sample.

	***n* = 169**
**SOCIODEMOGRAPHIC**	***n*** **(%)**
**Age groups**	
18–35	136 (80.5%)
36–68	33 (19.5%)
**Gender**	
Male	130 (76.9%)
Female	39 (23.1%)
**Area of origin**	
Africa	133 (78.7%)
America	8 (4.7%)
Asia	15 (8.8%)
Europe	13 (7.8%)
**Social status**	
Single	124 (73.4%)
Married	29 (17.2%)
Other (separated, divorced, and widowed)	16 (9.4%)
**Religious**	
Yes	154 (91.1%)
No	15 (8.9%)
**Occupational status**	
Unemployed	131 (77.5%)
Employed or student	38 (22.5%)
**Education**	
Illiterate	20 (11.8%)
Primary	41 (24.3%)
Lower/upper secondary	83 (49.1%)
Bachelor's degree or equivalent	20 (11.8%)
**Duration of stay in Italy (months)**	
≤ 12	37 (21.9%)
>12	132 (78.1%)
CLINICAL CHARACTERISTICS^*^	
Trauma-related disorders	51 (30.2%)
Mood disorders	38 (22.5%)
Anxiety disorders	12 (7.1%)
Adjustment disorder	12 (7.1%)
Borderline personality disorder	2 (1.2%)
Dysthymic disorder	2 (1.2%)
Insomnia disorder	7 (4.1%)
Substance use disorder	20 (11.8%)
Family history of mental disorders	18 (10.7%)

*No. and % do not add up to tot./100 owing to multiple responses in categories*.

* *Diagnoses related to the evaluation performed during intake*.

As shown in Table 2, the most frequent PTEs occurred during migration (A/DM) were serious injury (49.7%), imprisonment (41.4%), being close to death or lack of food and water (both 37.9%), and the lack of shelter (36.1%). The latter was the most common (31.4%) during the infancy period (I/PM), followed by the lack of food and water (30.2%) and rape or sexual abuse (18.3%). Overall, almost all migrants (94%) in the sample reported suffering a trauma during the migration process, whereas less than a half (43%) declared to having suffered a trauma during the infancy period. When we analyzed the relation between period of trauma occurrence and DSH, we did not find any statistically significant differences in the proportion of subjects with DSH: 28% of subjects who had a trauma injure themselves regardless of when their trauma occurred (data not shown).

**Table 2:** PTEs and period of occurrence (I/PM and/or A/DM).

**PTE** ^**a**^	**I/PM, *n* (%)**	**A/DM, *n* (%)**
Lack of food and water	51 (30.2%)	64 (37.9%)
No access to medical care	5 (3%)	45 (26.6%)
Lack of shelter	53 (31.4%)	61 (36.1%)
Imprisonment	– (–)	70 (41.4%)
Serious injury	1 (0.6%)	84 (49.7%)
Torture	1 (0.6%)	58 (34.3%)
Brainwashing	– (–)	7 (4.1%)
Rape or sexual abuse	31 (18.3%)	22 (13%),
Isolation from others	2 (1.2%)	40 (23.7%)
Being close to death	– (–)	64 (37.9%)
Forced separation	1 (0.6%)	52 (30.8%)
Murder of family or friend	4 (2.4%)	46 (27.2%)
Unnatural death of family/friend	5 (3%)	45 (26.6%)
Murder of stranger or strangers	1 (0.6%)	49 (29%)
Lost	1 (0.6%)	16 (9.5%)
Kidnapped	1 (0.6%)	35 (20.7%)
Combat situation	1 (0.6%)	57 (33.7%)

*PTEs, potentially traumatic events; I/PM, infancy/pre-migration; A/DM, adulthood/during migration*.

a *No. and % do not add up to tot./100 owing to multiple responses in categories*.

Of the sample, 26.6% declared a DSH episode in their lifetime (Table 3) with an age of onset of DSH ranging from 18 to 57 years, *M* = 29; *SD* = 8.65). To be engaged in DSH was most common in single (or alone) and unemployed migrants. Moreover, in subjects with post-traumatic stress symptoms, feelings of anxiety, hopelessness, low capability of resilience and SUI, and DSH behaviors were found to be more frequent.

**Table 3:** Characteristics of migrants who injure themselves.

	**All the samples** **(*N* = 169)**	**With DSH** **(*N* = 45, 26.6%)**	***p***
	***N***	***N* (%)**	
**SOCIODEMOGRAPHIC**			
**Age groups**			
18–35	136	37 (27.2%)	ns
36–68	33	8 (24.2%)	
**Gender**			
Male	130	33 (25.4%)	ns
Female	39	12 (30.8%)	
**Civil status**			
Single	124	33 (26.6%)	**<0.05**
Married	29	4 (13.8%)	
Other (separated, divorced, widowed)	16	8 (50.0%)	
**Religious**			
Yes	154	42 (27.3%)	ns
No	4	1 (25.0%)	
**Occupational status**			
Unemployed	131	43 (32.8%)	**<0.01**
Employed or student	38	2 (5.3%)	
**Education**			
Illiterate	20	5 (25.0%)	ns
Primary	41	13 (46.7%)	
Lower/upper secondary	83	21 (25.3%)	
Bachelor's degree or equivalent	20	4 (20.0%)	
**CLINICAL CHARACTERISTICS**			
**PTSD (PCL-5)**			
Yes	73	30 (41.1%)	**<0.001**
No	96	15 (15.6%)	
**Anxiety (SAS)**			
Yes	103	35 (34.0%)	**<0.01**
No	66	10 (15.2%)	
**Depression (SDS)**			
Yes	96	30 (31.3%)	ns
No	73	15 (20.5%)	
**Suicidal ideation (BSSI 1–5)**			
Yes	49	22 (44.9%)	**<0.001**
No	120	23 (19.2%)	
**Hopelessness (BHS)**			
Yes	110	38 (34.5%)	**<0.001**
No	59	7 (11.9%)	
**Low resilience (CD-RISC2)**			
Yes	105	37 (35.2%)	**<0.001**
No	64	8 (12.5%)	
**Lifetime substance use (yes)**			
Yes	20	7 (35.0%)	ns
No	135	32 (23.7%)	

*Bold values indicate statistical significance at p < 0.05, p < 0.01, and p < 0.001*.

*DSH, deliberate self-harm; PTSD, post-traumatic stress disorder; PCL-5, Posttraumatic Stress Disorder Checklist for DSM-5; SAS, Zung Self-Rating Anxiety Scale; SDS, Zung Self-Rating Depression Scale; BSSI, Beck Scale for Suicidal Ideation; BHS, Beck Hopelessness Scale; CD-RISC2, Connor-Davidson Resilience Scale 2*.

The results of univariate regression models for testing the association between each variable of interest and DSH are presented in Table 4. Anxiety, PTSD, SUI, hopelessness, and low resilience were significantly associated with a two-fold (or more) higher risk of engaging in DSH behaviors.

**Table 4:** Univariate log-binomial regression models for DSH behavior.

Variable^*^	**PR**	**[95% CI—inf]**	**[95% CI—sup]**	***p*-value**
Age-group (ref. 36+)	1.12	0.58	2.18	ns
Anxiety (SAS)	2.24	1.19	4.22	**<0.01**
PTSD (PCL-5)	2.63	1.53	4.51	**<0.001**
Depression (SDS)	1.52	0.89	2.61	ns
Suicidal ideation (BSSI-5)	2.34	1.45	3.79	**<0.001**
Hopelessness (BHS)	2.91	1.39	6.11	**<0.01**
Low resilience (CD-RISC2)	2.82	1.40	5.67	**<0.01**

*Bold values indicate statistical significance at p < 0.05*.

*DSH, deliberate self-harm; PR, prevalence ratio; SAS, Zung Self-Rating Anxiety Scale; PTSD, post-traumatic stress disorder; PCL-5, Posttraumatic Stress Disorder Checklist for DSM-5; SDS, Zung Self-Rating Depression Scale; CD-RISC2, Connor-Davidson Resilience Scale 2*.

* *“Absence” is considered as reference category*.

In Table 5, Model 1a shows the results of the multivariate regression model, where the effect of age, PTSD, and low resilience on DSH was tested. A strong association between PTSD (PR adj = 3.75 [1.77–7.94]), low resilience (PR adj = 3.91 [1.63–9.41]), and DSH was observed. Hopelessness (Model 1b) contributes to explaining part of the relation between PTSD and resilience with DSH. In fact, when including the variable hopelessness in the multivariate model, the effect of PTSD (PR adj = 2.21 [1.30–3.75]) and low resilience (PR adj = 2.32 [1.16–4.62]) on DSH decreased.

**Table 5:** Multivariate log-binomial regression models for DSH behavior.

Variable^*^	**PR adj**	**[95% CI—inf]**	**[95% CI—sup]**	***p*-value**
**Model 1a**				
Age group (ref. 36+)	1.16	0.44	3.04	ns
PTSD (PCL-5)	3.75	1.77	7.94	**<0.01**
Low resilience (CD-RISC2)	3.91	1.63	9.41	**<0.001**
Model 1b^**^				
Age group (ref. 36+)	1.21	0.67	2.19	ns
PTSD (PCL-5)	2.21	1.30	3.75	**<0.01**
Low resilience (CD-RISC2)	2.32	1.16	4.62	**<0.01**
Hopelessness (BHS)	2.01	0.95	4.22	ns

*Bold values indicate statistical significance at p < 0.05, p < 0.01, and p < 0.001*.

*DSH, deliberate self-harm; PR, prevalence ratio; PTSD, post-traumatic stress disorder; PCL-5, Posttraumatic Stress Disorder Checklist for DSM-5; CD-RISC2, Connor-Davidson Resilience Scale 2; BHS, Beck Hopelessness Scale; VIF, variance inflation factor*.

* *Where not explicit, “absence” is considered as reference category*.

** *VIF: Age group (1.031); PTSD (1.079); Resilience (1.066); Hopelessness (1.110)*.

## Discussion

This study aimed to explore the frequencies of DSH and its correlates among a nonclinical convenience migrant trauma-exposed subgroup. We have evaluated the relationship between DSH and some sociodemographic characteristics and clinical conditions. The results show high prevalence of DSH in the sample. We also found increased frequencies of PTSD, as well as depressive and other anxiety symptoms. The results show that trauma-related symptoms increase the risk of engaging in SIB. The data highlight that lower resilience resources correspond to an increase in DSH. Moreover, we also considered the feeling of hopelessness as a negative self-view characterized by rigid and persistently negative expectations about the future, which appears to have implications for post-traumatic-related symptoms. Epidemiological data suggest that trauma-exposed subjects who engage in DSH present a highly negative self-view (67). In addition, PTSD may impair the integrity of the self and result in negative expectations regarding the self and the world (68). Previous studies have provided empirical support for the existence of a relationship between traumatic experiences and self-injury (25) and have suggested that trauma-related symptoms may play an important role in the development and continuation of SIB. Other studies suggest that PTE rather than post-traumatic symptoms could be more strongly related to self-injury (48). Taken together, PTE and PTSD may play an important role in the development and continuation of DSH.

The issue of self-injury behavior is considered a pervasive public health burden that has received increasing attention from researchers (27, 69–71). In particular, DSH was considered one of the most important predictor factors for suicide among ethnic minorities (32). Our results indicate that DSH behaviors are associated with SAs and SUI. Data suggest that DSH may increase the risk of SUI and behavior.

A previous investigation conducted by Kalt et al. (72) in detentions centers showed a correlation between trauma and violence experienced during the migration processes and some psychopathological conditions like PTSD, psychosomatic symptomatology, SAs, and self-harm behavior. However, authors mention that these data should be considered with caution, since such behavior are not regularly reported. Moreover, there are still very few studies specifically focused on self-harm practices in asylum seekers and refugees in Europe (8).

According to Berry (73), in the context of transactional stress and coping models, migration processes invoke a vicious cycle of trauma and isolation that is influenced by an individual's resources to cope with stress, as well as by personal and social resources. Assessing trauma and trauma-related symptoms in migrants can contribute to a better understanding of the psychological correlates of self-injury, specifically enabling further examination of trauma symptoms as an underlying mechanism.

In particular, the exposure to catastrophic stress experiences related to persecution, war, or organized violence, circumstances where escape is unfeasible due to physical, psychological, environmental, or social constraints, may result in complex trauma or complex PTSD (CPTSD) (74, 75) (ICD-11; DSM-5). The construct of the CPTSD has drawn attention to the psychological consequences of interpersonal, prolonged, repeated, and extreme traumatization (74, 76). Many symptoms founded in our sample (e.g., negative self-views and negative expectations about the self and the world) could be related to CPTSD, which can be highly prevalent among immigrants and refugees [e.g., (77)]. It is important to evaluate PTSD and CPTSD, because trauma and complex trauma-related symptomatology may correlate with different conditions and require distinct interventions in mental healthcare with immigrant and refugees [e.g., (78)]. Such findings underlined the relevance of ensuring effective assessment of trauma-exposed migrants at the first stages, in order to reduce their psychological burden (79). In 2018, the increasing number of migrants in Europe has requested strategic actions like the “Migration and Health Programme” introduced by the WHO European Region to support the healthcare professionals in providing more prompt and robust responses to the needs of migrants.

Our study presents some limitations. First, the study was a cross-sectional design, and the sample is a nonclinical convenience sample, mainly made up of males, not representative of migrant populations. Therefore, migrants with different levels of severity of psychiatric symptomatology may not be included in our sample. Furthermore, this study did not have a large enough sample to allow the finding to be generalized to general migrant populations. Finally, our decision to dichotomize most of the variables to obtain more robust estimates and to make the interpretation easier may have produced an information loss about collected data, albeit modest.

The identification of DSH and/or SA would be particularly useful given the danger of these behaviors and the reduced life expectancy associated with them. Gaining a better understanding of what percentage of those who self-harm also attempt suicide, particularly in trauma-exposed migrants, is a necessary step in helping healthcare professionals to identify the phenomenon and to intervene.

The high prevalence of DSH in trauma-exposed migrants highlights the importance of routine assessments of these behaviors among this population. It is important that clinicians and physicians, including those in family medicine and primary care settings, are familiar with the association between PTE, PTSD, and DSH in order to implement programs focusing on prevention.

Finally, we would like to emphasize the need for future research to investigate the different prevalence of self-harm across cultures, as well as its cultural meanings and functions, in particular focusing on qualitative studies, which are better able to explore the subjective meanings of the asylum seekers' experiences and the deep functions of self-harm practices.

## Data Availability Statement

The datasets generated for this study are available on request to the corresponding author.

## Ethics Statement

The studies involving human participants were reviewed and approved by Comitato Etico Istituto Superiore della Sanità (Prot. PRE/17). The patients/participants provided their written informed consent to participate in this study.

## Author Contributions

MS conceived of the present study, participated in its design and coordination, and helped to revise the manuscript. FB devised the larger study from which this subset of data is drawn, wrote the paper, and helped to statistical analysis and to revise the manuscript. NN and CM conceived and designed the study, and wrote the paper. MV helped devise the larger study from which this subset of data is drawn, revised the manuscript, and conducted the statistical analysis. AP and ADN revise the statistical analysis and helped to draft the paper. All authors read and approved the final manuscript.

## Conflict of Interest

The authors declare that the research was conducted in the absence of any commercial or financial relationships that could be construed as a potential conflict of interest.

## Publisher's Note

All claims expressed in this article are solely those of the authors and do not necessarily represent those of their affiliated organizations, or those of the publisher, the editors and the reviewers. Any product that may be evaluated in this article, or claim that may be made by its manufacturer, is not guaranteed or endorsed by the publisher.
